# Myocardial Work Efficiency, A Novel Measure of Myocardial Dysfunction, Is Reduced in COVID-19 Patients and Associated With In-Hospital Mortality

**DOI:** 10.3389/fcvm.2021.667721

**Published:** 2021-06-14

**Authors:** Anum S. Minhas, Nisha A. Gilotra, Erin Goerlich, Thomas Metkus, Brian T. Garibaldi, Garima Sharma, Nicole Bavaro, Susan Phillip, Erin D. Michos, Allison G. Hays

**Affiliations:** ^1^Division of Cardiology, Department of Medicine, Johns Hopkins University School of Medicine, Baltimore, MD, United States; ^2^Division of Pulmonary and Critical Care Medicine, Department of Medicine, Johns Hopkins University School of Medicine, Baltimore, MD, United States

**Keywords:** echo, strain, COVID-19, non-invasive, ultrasoud diagnosis

## Abstract

**Background:** Although troponin elevation is common in COVID-19, the extent of myocardial dysfunction and its contributors to dysfunction are less well-characterized. We aimed to determine the prevalence of subclinical myocardial dysfunction and its association with mortality using speckle tracking echocardiography (STE), specifically global longitudinal strain (GLS) and myocardial work efficiency (MWE). We also tested the hypothesis that reduced myocardial function was associated with increased systemic inflammation in COVID-19.

**Methods and Results:** We conducted a retrospective study of hospitalized COVID-19 patients undergoing echocardiography (*n* = 136), of whom 83 and 75 had GLS (abnormal >−16%) and MWE (abnormal <95%) assessed, respectively. We performed adjusted logistic regression to examine associations of GLS and MWE with in-hospital mortality. Patients were mean 62 ± 14 years old (58% men). While 81% had normal left ventricular ejection fraction (LVEF), prevalence of myocardial dysfunction was high by STE; [39/83 (47%) had abnormal GLS; 59/75 (79%) had abnormal MWE]. Higher MWE was associated with lower in-hospital mortality in unadjusted [OR 0.92 (95% CI 0.85–0.99); *p* = 0.048] and adjusted models [aOR 0.87 (95% CI 0.78–0.97); *p* = 0.009]. In addition, increased systemic inflammation measured by interleukin-6 level was associated with reduced MWE.

**Conclusions:** Subclinical myocardial dysfunction is common in COVID-19 patients with clinical echocardiograms, even in those with normal LVEF. Reduced MWE is associated with higher interleukin-6 levels and increased in-hospital mortality. Non-invasive STE represents a readily available method to rapidly evaluate myocardial dysfunction in COVID-19 patients and can play an important role in risk stratification.

## Introduction

COVID-19, the disease caused by the novel coronavirus SARS-CoV2, carries high acute cardiovascular morbidity and mortality (1, 2). The mechanisms for cardiac injury are not fully understood, with hypotheses ranging from systemic inflammation due to cytokine release syndrome, angiotensin converting enzyme-2 mediated direct viral myocardial toxicity, autoimmune myocarditis, and sympathetic stress response (1, 3). Over 25% of hospitalized COVID-19 patients have acute cardiac injury as detected by elevated cardiac troponin, associated with greater in-hospital mortality (1, 3, 4). However, troponin alone has limited specificity and sensitivity in myocarditis and can also rise in acute respiratory distress syndrome (ARDS), another recognized complication of COVID-19 (5–7). Additionally, although multiple inflammatory pathways, such as interleukin-6 (IL-6), are implicated in myocardial injury in COVID-19, their effect on indices of cardiac function is unknown and a better understanding of the degree and determinants of myocardial function may improve risk stratification and lead to new therapeutic approaches (8–10).

Studies examining the degree of myocardial dysfunction in COVID-19 are limited, and assessment with cardiac imaging has been challenging due to exposure concerns to echocardiography staff. Thus, it is likely that the true prevalence of cardiac dysfunction is underreported (11). Speckle tracking echocardiography (STE) can rapidly quantify myocardial dysfunction (e.g., using global longitudinal strain [GLS]) with increased sensitivity compared with standard echocardiographic measures (12–14). More recently, a novel technique to measure LV function based on STE, global myocardial work (MW), was developed (15, 16). The advantage of MW [assessed by myocardial work index (MWI) and myocardial work efficiency (MWE)], is that it provides a more load independent measure of LV function by accounting for afterload; MW is also highly reproducible and adds incremental value to GLS in predicting adverse events (17).

Given the high mortality and severity of complications with COVID-19, we conducted a clinical cardiac imaging study in hospitalized COVID-19 patients with echocardiograms performed with the following aims: (1) to determine the prevalence and extent of myocardial dysfunction using STE (GLS and MWE), (2) to examine the association of myocardial dysfunction with in-hospital mortality, and (3) to investigate clinical and inflammatory biomarker risk factors associated with worsened subclinical myocardial dysfunction.

## Methods

### Study Design

This retrospective, single-center cohort study included 136 consecutive patients with confirmed COVID-19 who were hospitalized at Johns Hopkins Hospital and underwent clinically indicated transthoracic echocardiogram between March 25, 2020 and May 19, 2020, with follow-up completed by June 22, 2020. All echocardiograms were ordered by the patient's clinical care team. The study was approved by the Johns Hopkins Institutional Review Board and informed consent was waived per IRB guidelines.

### Clinical Data

Patient characteristics, including demographics, medical history, clinical presentation, laboratory testing, and clinical outcomes were extracted from the electronic medical record. Initial values after admission for the following serum biomarkers were collected: cardiac troponin I, IL-6, C-reactive protein (CRP), ferritin, fibrinogen, and d-dimer. In- hospital all-cause mortality during index hospitalization was ascertained from electronic medical records through the end of follow-up. Two separate investigators independently reviewed the data.

### Transthoracic Echocardiography

#### Conventional 2D Echocardiographic Analysis

Bedside transthoracic echocardiographic (TTE) examinations were performed by experienced sonographers using Vivid^™^ E95 ultrasound system (GE Vingmed Ultrasound; Horten, Norway). Both standard 2D and Doppler echocardiography were acquired. Measurements including LV, right ventricular (RV) parameters and diastology were performed by a dedicated research sonographer based on the American Society of Echocardiography (ASE) guidelines (18, 19). To limit exposure to patients and staff, measurements that were not essential, including STE analyses, were performed offline, removed from the patient's room, and limited studies were performed according to COVID-19 specific imaging guidelines (20).

#### Speckle Tracking Echocardiography Analysis

STE analyses were conducted according to ASE recommendations in a subset of TTEs that were (1) deemed to be of fair quality or greater for subendocardial image visualization by two independent readers and (2) in a patient free of atrial or ventricular arrhythmias at the time of exam (*n* = 83) (18). Two-dimensional images from the apical four-chamber, two-chamber, and long-axis views were acquired with frame rates between 50 and 80 frames/s to enable GLS. GLS was quantified using semiautomated analysis software (EchoPAC version 202; GE Vingmed Ultrasound). The automated algorithm traces and tracks the LV myocardium, with manual adjustments made when appropriate, and the software calculates GLS from the weighted average of the peak systolic longitudinal strain of all segments using the 17 segment model. GLS is quantified as a negative number with cutoff as −18%, and more negative as normal for this system, but based on prior literature supporting use of a cutoff of −16% as the threshold for normal, analyses were conducted with > −16% as the cutoff for normal (21–25). Tracking quality was assessed by the operator and over-ridden in segments with two or fewer rejected regions where the operator deemed tracking quality to be acceptable. Images were analyzed by two independent observers blinded to clinical data on a dedicated offline research workstation. Intraobserver and interobserver variability of STE measures, specifically MWE, were assessed by intra- class correlation coefficient (individual ICC of 0.994 and average ICC of 0.997 for intraobserver and 0.992 and 0.995 for interobserver, respectively), and Bland-Altman analysis (all differences in measurements within ±1 SD). The time between intraobserver measurements was 1 day.

### Myocardial Work

**Figure 1:**
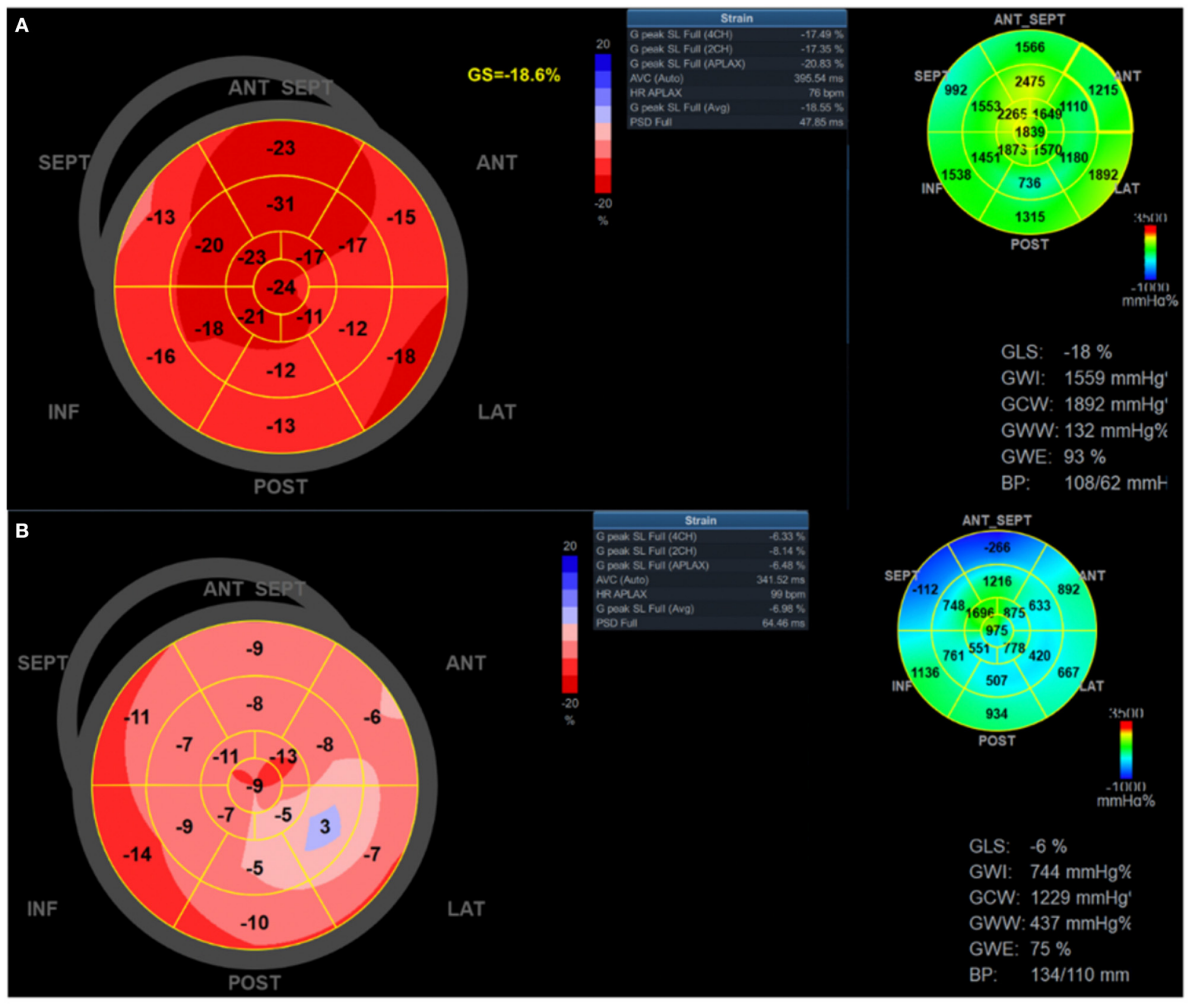
Global longitudinal strain and myocardial work efficiency measurement in patients with COVID-19. Global longitudinal strain and myocardial work index bull's eye mapping for two patients with COVID-19. **(A)** representative patient with relatively normal strain and myocardial work; **(B)** representative patient with severely reduced global longitudinal strain (apical predominant), myocardial work index, and work efficiency. ANT, anterior; ANT SEPT, anterospetal; APLAX, apical long axis; AVC, aortic valve closure; CH, chamber; GS, global strain; HR, heart rate; INF, inferior; LAT, lateral; POST, posterior; PSD, peak systolic dispersion; SEPT, septal; SL, strain length.

Myocardial work (MW) was determined from non-invasive LV pressure-strain analysis, which has previously been described and validated (26, 27). MW is calculated as the area of the pressure-strain loop, similar in concept to deriving LV stroke work using pressure volume loops invasively. In this technique, pressures are assessed using brachial systolic pressure and valvular event timing and strain measured with STE (15, 16). MW indices were calculated with the same software as above to evaluate LV performance by incorporating afterload determination using blood pressure; this provides a more load-independent measure compared with GLS (27). Blood pressure was measured by sphygmomanometry at the time of the echocardiogram immediately before acquiring images for STE. The MW software then constructs a non-invasive LV pressure curve adjusted according to the duration of isovolumic and ejection phases defined by the timing of aortic and mitral valve opening and closing events (28). Global MW was quantified by calculating the rate of regional shortening by differentiation of the strain tracing and multiplying by instantaneous LV pressure (estimated) integrated over time. During LV ejection time, segments were analyzed for wasted work and constructive work, with global values determined as the averages of all segmental values (see example Figure 1). The following parameters were acquired using EchoPAC software: Global MW index (MWI, mmHg%) defined as the area within the global LV pressure-strain loop and global MW efficiency (MWE, %), defined as constructive MW divided by the sum of constructed work and wasted work, expressed as a percentage. Abnormal MWE was defined as <95%, consistent with other studies (16). For myocardial work, MWE was chosen as the primary variable of interest as it provides a comprehensive assessment of the ratio between constructive work performed by the LV and the sum of both wasted and constructive work, and has previously shown to have prognostic value in other populations (29–31).

### Statistical Methods

Descriptive statistical analyses were performed for clinical and echocardiographic parameters. Continuous variables are presented as mean ± standard deviation (normally distributed variables) or median (IQR) (non-normally distributed variables). Differences between groups were compared using parametric two-sample Student's *t*-test or non-parametric Mann–Whitney *U*-test. Categorical variables are presented as number (%) and groups compared using Chi-squared test. For relevant analyses, normal LVEF was defined as >50%.

We then performed unadjusted and adjusted logistic regression to estimate the odds of mortality with either GLS or MWE as the primary independent variable of interest, analyzed continuously. Covariates included were clinical characteristics (age, sex, diabetes, and hypertension) and echocardiographic measurements, selected one at a time for addition to the model as the primary covariate of interest (LVEF, GLS, MWE, TAPSE, RVSP, TR peak velocity, and E/E′). Clinical covariates selected for inclusion in the adjusted models were chosen based on prior literature suggesting possible confounding, and included age, sex, history of hypertension, and diabetes (32–36). Model 1 included the echocardiographic covariate of interest, adjusted for age and sex. Model 2 included the echocardiographic covariate of interest, adjusted for age, sex, diabetes, and hypertension. All variables for logistic regression were analyzed as continuous variables.

To further understand the incremental value of STE analysis over standard echocardiographic LVEF assessment for mortality prediction, we performed subgroup analyses in patients with normal (>50%) or abnormal (<50%) LV EF. We also performed subgroup analyses in patients with the presence or absence of acute respiratory distress syndrome (ARDS). A *p* ≤ 0.05 was considered significant.

Last, for a subset of the cohort, linear regression was then performed to evaluate inflammatory markers (divided into tertiles given non-normal distribution) as predictors of MWE. Values within each tertile are included in the supplement. These markers included IL6, troponin, ferritin, C-reactive protein (CRP), d-dimer, and fibrinogen. Missing data were considered to occur at random, and patients with missing inflammatory data were not included in this analysis.

## Results

### Clinical Characteristics of Patients Undergoing Echocardiogram

Median time of symptom duration prior to admission was 6 days (3–8 days). Median time to echocardiogram after admission was 4 days (2–8 days) and median overall time of admission was 16.5 days (9–31 days).

Clinical characteristics of hospitalized patients with COVID-19 who had echocardiogram performed are shown in Table 1 (*n* = 136). The mean age was 62 years, 79 (58%) were men and 63 (47%) African American. Approximately 63% of patients required mechanical ventilation, 57% were diagnosed with ARDS and 53% had shock (septic, distributive, cardiogenic or otherwise) (Table 1).

**Table 1:** Comparison of clinical characteristics and echocardiographic parameters in the cohort of hospitalized patients with COVID-19 and subgroups with normal vs. abnormal global longitudinal strain (GLS) and myocardial work efficiency (MWE).

**Variables**	**Overall cohort** ***N* = 136**	**Normal GLS** ***N* = 44**	**Abnormal GLS** ***N* = 39**	***p*-value**	**Normal MWE** ***N* = 16**	**Abnormal MWE** ***N* = 59**	***p*-value**
Age, years	62.4 ± 13.9	61.9 ± 13.4	63.4 ± 14.4	0.614	55.2 ± 16.5	64.3 ± 13.1	**0.023**
Male	79 (58%)	27 (61%)	22 (56%)	0.647	13 (81%)	32 (53%)	**0.039**
Race				0.347			0.082
White	34 (25%)	10 (23%)	5 (13%)		3 (19%)	12 (21%)	
African American	63 (47%)	20 (45%)	23 (61%)		5 (31%)	33 (57%)	
Other	37 (27%)	14 (32%)	10 (26%)		8 (50%)	13 (22%)	
Body mass index, kg/m^2^	30.0 (26.4–35.8)	27.8 (25.6–31.3)	31.4 (26.5–38.4)	**0.017**	27.7 (25.7–31.8)	28.7 (25.7–34.5)	0.544
**Comorbidities**							
Hypertension	97 (72%)	29 (66%)	30 (77%)	0.269	7 (44%)	46 (78%)	**0.008**
Diabetes mellitus	55 (41%)	12 (27%)	20 (51%)	**0.025**	1 (6%)	29 (49%)	**0.002**
Coronary artery disease	20 (15%)	4 (9%)	8 (21%)	0.140	0 (0%)	10 (17%)	0.077
Heart failure	20 (15%)	2 (5%)	12 (31%)	**0.001**	0 (0%)	12 (20%)	**0.049**
**Clinical presentation**							
Heart rate, beats per min	99 ± 20	97 ± 17	103 ± 21	0.151	95 ± 18	100 ± 20	0.392
Systolic blood pressure, mmHg	129 ± 25	129 ± 24	134 ± 24	0.368	126 ± 27	132 ± 23	0.343
Diastolic blood pressure, mmHg	71 ± 16	71 ± 16	74 ± 15	0.389	74 ± 16	71 ± 16	0.546
**Laboratory measurements**							
White blood cell count, K/cu mm	6.7 (5.0–9.3)	6.4 (4.6–8.7)	6.0 (4.8–8.3)	0.773	6.4 (4.8–9.0)	6.4 (4.8–9.1)	0.946
Absolute lymphocyte count, K/cu mm	0.6 (0.1–1.1)	0.6 (0.1–1.0)	0.5 (0.0–1.3)	0.794	0.7 (0.0–1.2)	0.7 (0.03–1.2)	0.992
D-dimer, mg/L	2.0 (0.8–5.3)	2.0 (0.8–4.6)	2.2 (0.9–7.3)	0.433	2.0 (0.4–4.7)	2.2 (0.9–4.5)	0.213
Interleukin-6, pg/ml	130 (51–409)	86 (32–167)	164 (69–815)	**0.034**	114 (47–422)	125 (45–406)	0.695
CRP, mg/dl	15.3 (4.9–34.7)	11.7 (3.3–20.5)	13.7 (5.1–37.7)	0.410	4.9 (2.3–15.3)	15 (6.6–34.3)	**0.009**
Ferritin, ng/ml	735 (395–1,424)	737 (427–1,130)	800 (402–2,898)	0.525	830 (289–1,677)	719 (412–1,125)	0.897
Fibrinogen, mg/dl	596 (445–703)	737 (427–1,130)	800 (402–2,898)	0.695	568 (463–729)	597 (457–722)	0.694
Pro-BNP, pg/ml	422 (157–1,956)	242 (99–589)	564 (164–3,992)	**0.044**	176 (70–385)	392 (164–2,611)	**0.032**
Troponin I, ng/ml	0.03 (0.03–0.05)	0.03 (0.03–0.03)	0.03 (0.03–0.08)	0.454	0.03 (0.03–0.03)	0.03 (0.03–0.05)	0.305
**Clinical events**							
Shock	72 (53%)	17 (39%)	23 (59%)	0.064	4 (25%)	30 (51%)	0.065
Mechanical ventilation	86 (63%)	22 (50%)	26 (67%)	0.125	5 (31%)	38 (64%)	**0.017**
ARDS	78 (57%)	19 (43%)	25 (64%)	0.057	5 (31%)	32 (54%)	0.103
DVT or PE	31 (23%)	8 (18%)	8 (21%)	0.788	3 (19%)	12 (20%)	0.888
Death	25 (19%)	7 (16%)	8 (21%)	0.620	2 (12%)	9 (16%)	0.764
**Echocardiographic parameters**							
LA volume, ml	44 (35–71)	41 (29–45)	48 (39–95)	**0.046**	39.5 (28–42)	47 (39–55)	0.222
LVEDD, cm	4.2 (3.7–4.8)	4.1 (3.8–4.6)	4.3 (3.4–4.9)	0.378	4.4 (3.8–4.9)	4.1 (3.5–4.7)	0.276
LVEF, %	62 (52–62)	62 (57–64)	55 (40–62)	** <0.001**	62 (62–64)	57 (50–62)	**0.011**
Normal LVEF (>50%)	109 (81%)	43 (64%)	24 (36%)	** <0.001**	16 (100%)	45 (74%)	**0.031**
RVEDD, cm	3.6 ± 0.7	3.4 ± 0.6	3.6 ± 0.7	0.224	3.4 ± 0.7	3.6 ± 0.6	0.225
Normal RV function	63 (81%)	22 (85%)	18 (72%)	0.274	12 (92%)	24 (73%)	0.147
TAPSE, cm	1.8 ± 0.4	2.0 ± 0.4	1.7 ± 0.4	**0.005**	2.1 ± 0.3	1.8 ± 0.4	**0.003**
RVSP, mmHg	37 (30–50)	37 (29–48)	34 (32–53)	0.742	31 (30–33)	37 (29–49)	0.288
Mean PAP, mmHg	34 ± 12	35 ± 9	34 ± 11	0.754	27 ± 12	35 ± 9	0.087
Peak TR gradient, mmHg	31 (25–42)	32 (25–42)	31 (25–40)	0.899	29 (25–38)	31 (24–43)	0.832
PCWP, mmHg	14 (10–18)	13 (9–17)	12 (9–16)	0.820	12 (10–16)	15 (12–21)	0.422
E/E'	10 (8–13)	10 (7–12)	9 (7–13)	0.665	9 (7–11)	9 (7–13)	0.561
GLS, %	−16.1 ± 4.3	−19.2 ± 2.4	−12.6 ± 3.0	** <0.001**	−19.7 ± 3.1	−15.5 ± 4.1	** <0.001**
MWI, mmHg%	1,412 ± 425	1,579 ± 362	1,227 ± 417	** <0.001**	1,723 ± 399	1,331 ± 396	** <0.001**
MWE, %	92 (87–94)	94 (91–95)	89 (82–92)	** <0.001**	96 (95–96)	91 (86–93)	** <0.001**

*Categorical variables are presented as number (%) and continuous variables are presented as mean ± standard deviation or median (interquartile range)*.

*CRP, C-reactive protein; Pro-BNP, N-terminal pro-hormone B-type natriuretic peptide; ARDS, acute respiratory distress syndrome; DVT, deep venous thrombosis; PE, pulmonary embolism; LA, left atrium; LVEDD, left ventricular end diastolic diameter; LVEF, left ventricular ejection fraction; GLS, global longitudinal strain; RVEDD, right ventricular end diastolic diameter; TAPSE, tricuspid annular plane systolic excursion; RVSP, right ventricular systolic pressure; PAP, pulmonary artery pressure; TR, tricuspid regurgitation; GLS, global longitudinal strain; MWI, myocardial work index; MWE, myocardial work efficiency*.

*The bold values represent significant p-values, with significant defined as <0.05*.

The cohort of patients with echocardiograms performed was comparable to the subset of patients with GLS and MWE measured (Table 1). The majority of patients (81%) undergoing echocardiogram had normal LV systolic function by LVEF measurement. Follow-up (discharged as alive or deceased) was complete for 131/136 patients, while 5/136 (3.7%) were administratively censored (still admitted at the time of analysis).

### Clinical and Echocardiographic Characteristics for Patients With Global Longitudinal Strain Assessed

Among the patients with GLS performed (*n* = 83), 44 patients had normal GLS and 39 (47%) had abnormal GLS (Table 1). There were no significant differences in age, sex, race, or history of hypertension or CAD between patients with and without abnormal GLS. There was higher prevalence of diabetes mellitus in the abnormal compared with normal GLS group (51 vs. 27%, *p* = 0.025). Body mass index (BMI) was significantly higher in patients with abnormal compared with normal GLS (median 31.4 vs. 27.8 kg/m^2^, *p* = 0.017). Patients with abnormal GLS had lower LVEF (55 vs. 62%, *p* < 0.001), and lower TAPSE (1.7 vs. 2.0 cm, *p* = 0.005) when compared with those with normal GLS.

Among the inflammatory markers, interleukin-6 was higher among patients with abnormal GLS [median 164 (69–815)] compared with normal GLS [median 86 (32–167)], *p* = 0.034. All other inflammatory markers were not significantly different (Table 1). The value ranges of each inflammatory marker per tertile are presented in Supplementary Table 1.

### Clinical and Echocardiographic Characteristics for Patients With Myocardial Work Efficiency Assessed

Among the subgroup of patients with myocardial work imaging performed (*N* = 75), abnormal MWE (defined as <95%) was present in the majority (59/75, 79%). There were no significant differences in demographics or clinical presentation between patients with normal vs. abnormal MWE (Table 1). A history of hypertension was more common among patients with abnormal MWE compared with normal MWE (78 vs. 44%, *p* = 0.008), as was a prior history of diabetes (29 vs. 1%, *p* = 0.002). Patients with abnormal MWE compared with those with normal MWE had lower LVEF (57 vs. 62%, *p* = 0.011), and lower TAPSE (1.8 vs. 2.1 cm, *p* = 0.003).

**Figure 2:**
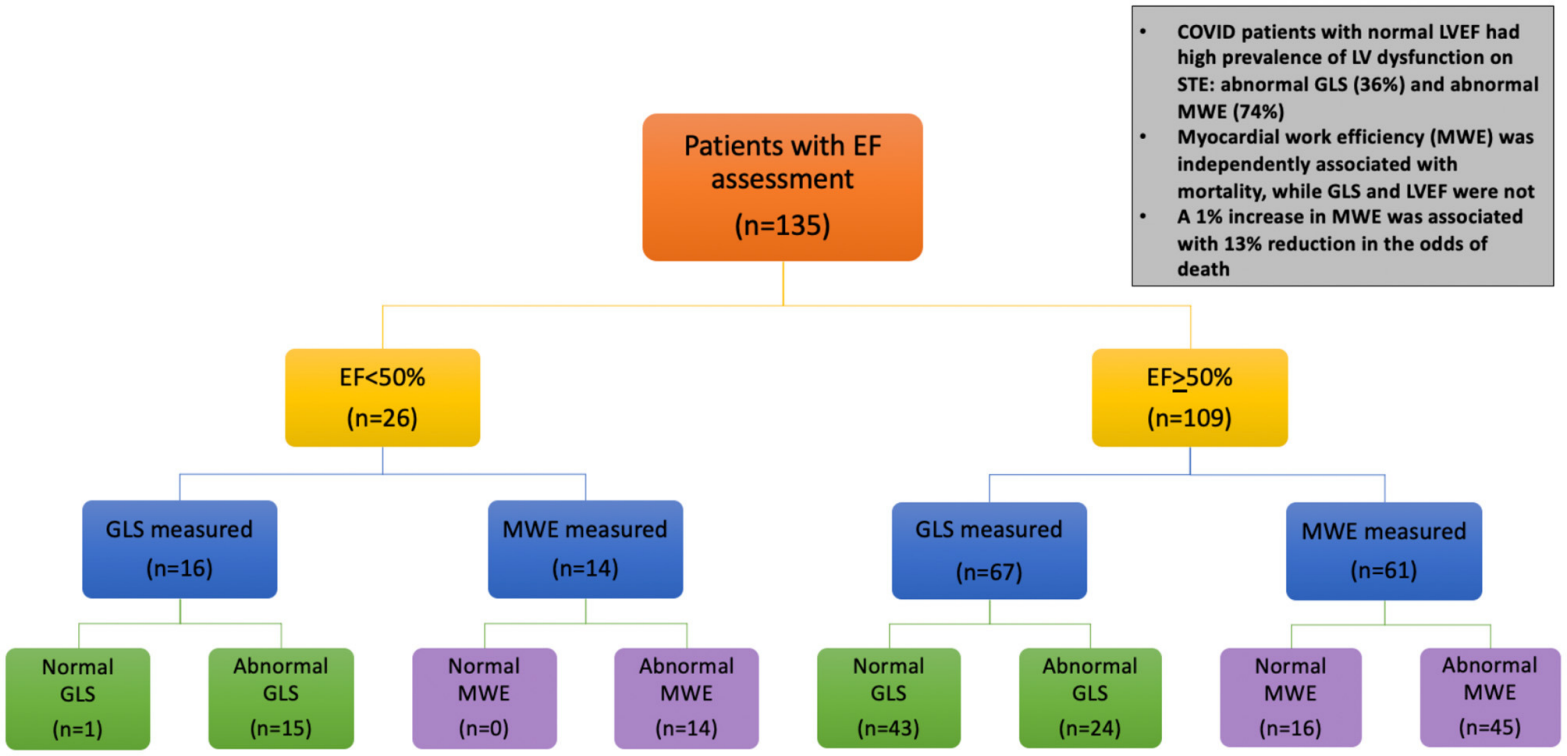
Echocardiogram evaluation and main findings in hospitalized patients with COVID-19. Flow diagram of the study shows number of patients undergoing echocardiogram, including with speckle tracking technique for strain measures. GLS, global longitudinal strain; LVEF, left ventricular ejection fraction; MWE, myocardial work efficiency. Abnormal GLS is defined as ≤16% (the absolute value of −16%). Additional abbreviations in Figure 1.

Among patients with normal LVEF (*n* = 67), a high percentage had evidence of subclinical myocardial dysfunction using STE: 36% had abnormal GLS (GLS>−16%) and 74% had abnormal MWE (MWE <95%) (Figure 2).

#### Association of Clinical Characteristics and Speckle Tracking Echocardiography Measurements With Mortality

During hospital admission, 25 (19%) of patients experienced in-hospital death. No clinical characteristics were independently associated with mortality in univariate analysis. MWE was the only echocardiographic parameter independently associated with mortality [unadjusted OR 0.92 (95% CI 0.85–0.999), *p* = 0.048]. In adjusted Models 1–2, MWE remained associated with mortality, with the strongest association in Model 2 [OR 0.87 (95% CI 0.78–0.97), *p* = 0.009] (Table 2), suggesting that a 1% increase in MWE was associated with 13% lower odds of death.

**Table 2:** Association of each echocardiographic parameter with mortality in hospitalized patients with COVID-19.

	**Unadjusted**	**Model 1** **(age and sex)** **odds ratio (95% CI)**	**Model 2** **(age, sex, diabetes, hypertension)** **odds ratio (95% CI)**
LVEF	1.00 (0.96–1.03) *P* = 0.248	1.00 (0.96–1.04)*P* = 0.934	1.00 (0.96–1.04) *P* = 0.918
GLS	1.07 (0.94–1.22) *P* = 0.287	1.08 (0.94–1.23)*P* = 0.287	1.15 (0.98–1.35) *P* = 0.089
MWE	0.92 (0.85–0.999) ***P*** **=** **0.048**	0.90 (0.81–0.98)***P*** **=** **0.021**	0.87 (0.78–0.97) ***P*** **=** **0.009**
TAPSE	0.43 (0.11–1.71) *P* = 0.230	0.41 (0.10–1.74)*P* = 0.228	0.30 (0.06–1.45) *P* = 0.135
RVSP	1.04 (1.00–1.09) *P* = 0.051	1.04 (1.00–1.09)*P* = 0.073	1.04 (1.0–1.09) *P* = 0.081
TR peak velocity	1.03 (0.99–1.07) *P* = 0.182	1.03 (0.98–1.07)*P* = 0.219	1.03 (0.98–1.07) *P* = 0.235
E/E'	0.97 (0.91–1.05) *P* = 0.459	0.96 (0.87–1.06)*P* = 0.392	0.97 (0.90–1.05) *P* = 0.498

*The bold values represent significant p-values, with significant defined as <0.05*.

Additional subgroup analyses performed to confirm the relationship of MWE and mortality showed similar findings. Among patients with normal LVEF, higher MWE was again independently inversely associated with death [unadjusted OR 0.89 (95% CI 0.78–1.00), *p* = 0.050]. MWE was also associated with in-hospital death after adjusting for age and sex [aOR 0.85 (95% CI 0.74–0.99), *p* = 0.038]. GLS was not associated with death in adjusted or unadjusted analysis. No echocardiographic parameter (LVEF, GLS, or MWE) was associated with mortality in subgroup analyses of patients with and without ARDS (Supplementary Table 2).

**Figure 3:**
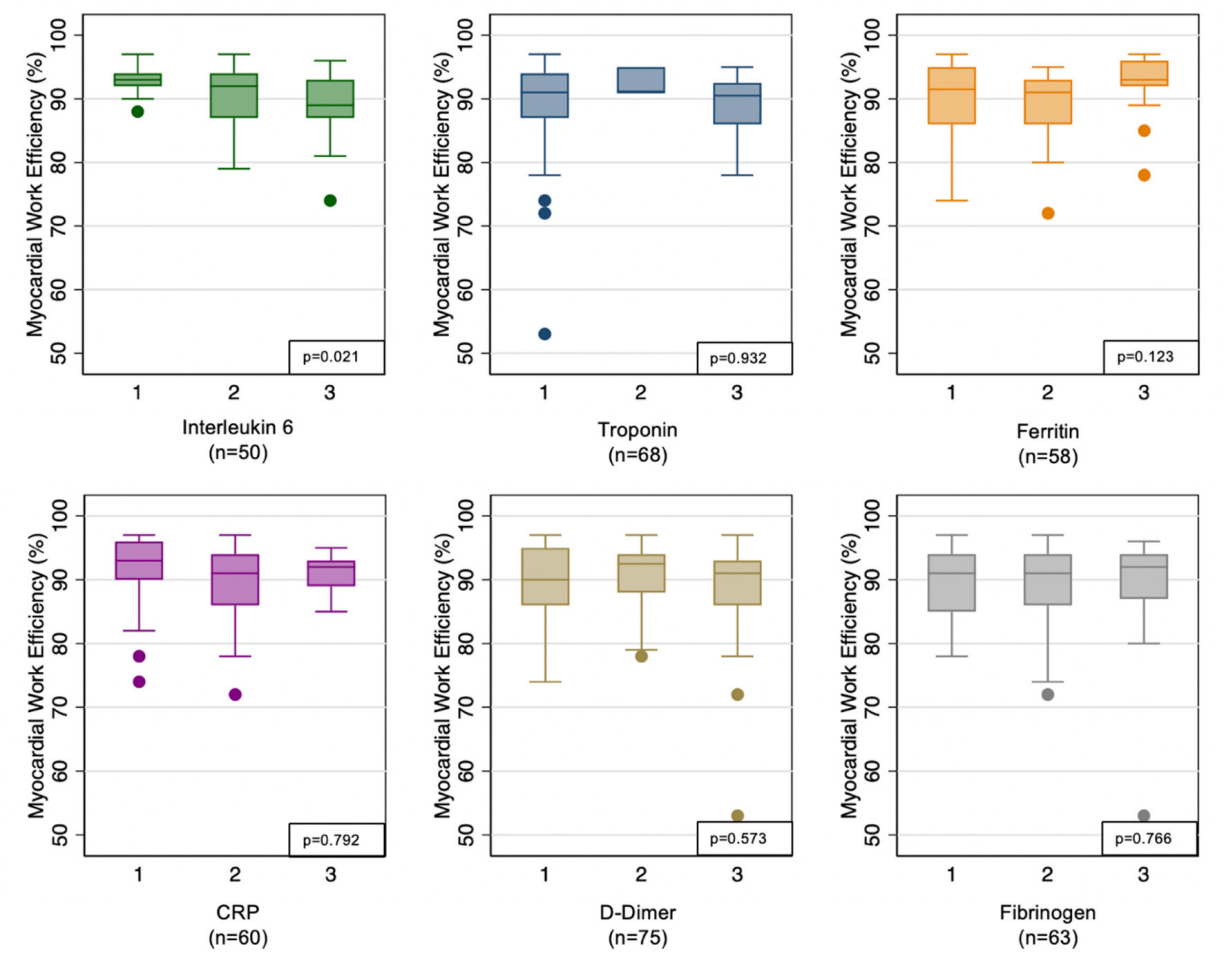
Association of myocardial work efficiency with inflammatory markers. Inflammatory markers are analyzed by tertile of each marker given non-normal distribution.

As MWE was the only echocardiographic parameter associated with mortality, we then evaluated systemic inflammatory markers as predictors of abnormal MWE in a subset of patients with available inflammatory marker data. We observed that MWE was 2.04% lower per higher tertile of IL-6 level (*p* = 0.021), indicating that greater degree of inflammation reflected by IL-6 levels were associated with worse myocardial function as measured using MWE. All other inflammatory markers tested were associated with no difference in MWE (Figure 3).

## Discussion

We report that subclinical cardiac dysfunction measured by GLS and MWE on STE is common in hospitalized COVID-19 patients with clinically indicated echocardiograms performed. To our knowledge, this is one of the first reports characterizing novel echocardiographic indices of myocardial dysfunction (GLS and the newer imaging parameter MWE) in hospitalized patients with COVID-19. We report several unique findings in our population: (1) Subclinical myocardial dysfunction is prevalent among COVID-19 patients even in the setting of normal LVEF, especially in those with traditional cardiovascular risk factors, (2) lower, more abnormal MWE, which is a sensitive measure of load independent myocardial dysfunction, is associated with greater in-hospital mortality, and (3) higher level of the inflammatory marker, IL-6, is predictive of lower MWE. Importantly, the finding of the association of MWE with mortality held true even after analyzing patients with normal LVEF, suggesting the prognostic benefit of MWE over LVEF and supporting use of MWE in addition to LVEF for hospitalized patients with COVID-19.

### Speckle Tracking Echocardiography for the Detection of Subclinical Myocardial Dysfunction in COVID-19 Patients

Both GLS and MWE are sensitive measures of LV function and cardiac injury, and the current study is among the first to characterize these indices in the setting of acute COVID-19 (37, 38). Compared with LVEF, GLS improves risk stratification, enhances disease classification, and may guide the treatment approach in asymptomatic patients with subclinical LV dysfunction (14, 38). Both GLS and MWE measurements are validated, reproducible, and do not require additional imaging beyond standard TTE, reducing potential additional provider exposure during image acquisition. Prior studies have consistently demonstrated reduced GLS despite a preserved LVEF among patients at increased risk for cardiac injury and dysfunction (39). MWE is a newer load-independent measure that permits both global and regional ventricular mechanics to be analyzed through the relationship between myocardial contractility and LV pressure (15). A previous study showed that non-invasive indices of myocardial work are more sensitive than GLS for the detection of significant CAD in patients with normal regional wall motion and preserved LVEF (17). The present study supports these prior findings, as abnormal MWE was even more prevalent than abnormal GLS (79 vs. 46% of patients). Additionally, patients with abnormal STE indices were more likely to have cardiovascular risk factors than those with normal indices, even among those with normal LVEF.

### Myocardial Work Efficiency and Mortality

While recent data has suggested high incidence of acute cardiac injury by troponin levels in COVID-19, investigations into the extent and implications of myocardial dysfunction on adverse outcomes such as death are limited (1, 7, 13, 40–42). In the present study, in a cohort with comparable in-hospital mortality to prior studies in COVID-19, we demonstrate the ability of MWE to predict mortality while GLS and LVEF did not. Prior studies suggest that the amount of myocardial work is related to uptake of fluro-deoxy-glucose at myocardial positron emission tomography scan, suggesting a relationship between myocardial work efficiency and metabolism (27). It is possible that impaired MWE may be related to derangements in myocardial metabolism that can occur in the setting of increased systemic inflammation.

Based on these findings, it is possible that STE measures of subclinical LV dysfunction may provide incremental value to standard echo measures in patients with COVID-19. Given the acuity of presentation and cardiovascular complications of COVID-19, a better understanding of the extent of myocardial injury and dysfunction early in the disease course may help triage at risk patients and implement early interventions aimed at reducing mortality.

### Systemic Inflammation and Cardiac Dysfunction

Although recent studies have aimed to describe pathophysiologic processes leading to RV strain and dilation in acute COVID-19 (43, 44), LV dysfunction and particularly subclinical dysfunction on STE, have not been as well-investigated. Studies suggest that increased systemic inflammation and impaired immune function may play a role (6, 7). Potential causes of myocardial dysfunction include myocarditis, ischemic injury (caused by microvascular dysfunction or epicardial CAD), stress cardiomyopathy or cytokine release syndrome (45). Autopsy studies of severe COVID-19 disease suggest there can also be direct viral-induced injury of multiple organs, including the heart (46). However, the relative contribution and determinants of myocardial dysfunction have not been well-characterized, partially due to limited ability to obtain widespread cardiac testing in these patients. Given these limitations, the true prevalence of cardiac dysfunction has likely been underreported thus far, and is mainly limited to case reports (6, 7).

In our study, patients underwent echocardiography at a median 4 days after hospital admission and 6 days of symptom onset, suggesting that impaired GLS and MWE occur early in COVID-19 during the systemic inflammatory response, and cannot entirely be explained by a more chronic myocardial process such as fibrosis. In addition, COVID-19 patients with LV dysfunction on STE had more obesity, which is a pro-inflammatory state that initiates oxidative stress and adversely affects immune function, leading to cardiac injury (40, 47, 48). Finally, although inflammatory pathways have been implicated in myocardial injury related to COVID-19, their effect on important indices of cardiac function has not been well-characterized. In the present study, we show that subclinical myocardial dysfunction is related to the degree of systemic inflammation measured by IL-6. IL-6 has previously been shown to act as a key cytokine in producing downstream effects resulting in organ damage, including reduced myocardial contractility (49–51).

Additionally, IL-6 levels in the setting of COVID-19 have been reported to be elevated in several studies and have been shown to correlate with mortality (52–54). Our study, along with these prior studies, supports a potential role of IL-6 and heightened inflammation in mediating myocardial dysfunction, thereby increasing risk of death. Of note, we did not find a similar relationship with troponin and myocardial dysfunction, likely related to the primarily normal-range troponin values for the majority of patients.

By characterizing subclinical myocardial dysfunction using STE, the present study provides incremental knowledge, linking increased systemic inflammation (by IL-6 levels) to the pathophysiology of myocardial injury and dysfunction in COVID-19.

### Limitations

The main limitation of this study is the relatively small sample size and retrospective cohort study design. Larger prospective studies are needed to further explore these novel echocardiographic parameters (GLS and MWE) with regard to cardiovascular mortality and other clinically meaningful outcomes in COVID-19 disease. Also, not all hospitalized COVID-19 patients underwent echocardiogram and STE, which could result in selection bias and inability to detect true prevalence of abnormal GLS or MWE among COVID-19 patients. Lastly, a minority of patients with GLS and MWE performed did not have all inflammatory markers tested clinically, thus limiting the analyses.

## Conclusions

In summary, sensitive indices of LV dysfunction, GLS and MWE, measured with STE are abnormal in a substantial portion of hospitalized COVID-19 patients who underwent echocardiograms, even in those with normal LVEF. Impaired MWE is independently associated with in-hospital mortality in COVID-19 patients. Higher IL-6 levels are associated with reduced MWE, providing a possible pathophysiologic link between increased inflammation and adverse outcomes in COVID-19. Based on these findings, it is possible that STE measures of subclinical LV dysfunction may provide incremental value to standard echocardiographic measures in patients with COVID-19. Given the acuity of presentation and cardiovascular complications of COVID-19, a better understanding of the extent of myocardial injury and dysfunction early in the disease course may help triage at risk patients and implement early interventions aimed at reducing mortality. Further longitudinal studies are needed to investigate persistence of impaired cardiac function in the setting of COVID-19.

## Data Availability Statement

The raw data supporting the conclusions of this article will be made available by the authors, without undue reservation.

## Ethics Statement

The studies involving human participants were reviewed and approved by Johns Hopkins IRB. Written informed consent for participation was not required for this study in accordance with the national legislation and the institutional requirements.

## Author Contributions

AM, NG, EG, and AH drafted the manuscript. BG, TM, GS, SP, and NB edited the manuscript. AM, NG, EG, and NB collected the data. AM analyzed the data and performed statistical analysis. All authors contributed to the article and approved the submitted version.

## Conflict of Interest

The authors declare that the research was conducted in the absence of any commercial or financial relationships that could be construed as a potential conflict of interest.

## Abbreviations

COVID-19: SARS-CoV2
ARDS: acute respiratory distress syndrome
GLS: global longitudinal strain
MW: myocardial work
MWI: myocardial work index
MWE: myocardial work efficiency
LV: left ventricular
RV: right ventricular
EF: ejection fraction
LVEDD: left ventricular end diastolic diameter
RVEDD: right ventricular end diastolic diameter
TAPSE: tricuspid annular plane systolic excursion
TR: tricuspid regurgitation
STE: speckle tracking echocardiography
ASE: American Society of Echocardiography
BMI: body mass index
CRP: C-reactive protein
IL-6: interleukin 6
Pro-BNP: N-terminal pro-hormone B-type natriuretic peptide.

## References

[1] Guo T Fan Y Chen M Wu X Zhang L He T. Cardiovascular implications of fatal outcomes of patients with coronavirus disease 2019 (COVID-19). JAMA Cardiol. (2020) 5:811–8. 10.1001/jamacardio.2020.1017 32219356 PMC7101506

[2] Shi S Qin M Shen B Cai Y Liu T Yang F. Association of cardiac injury with mortality in hospitalized patients with COVID-19 in Wuhan, China. JAMA Cardiol. (2020) 5:802–10. 10.1001/jamacardio.2020.0950 32211816 PMC7097841

[3] Hoffmann M Kleine-Weber H Schroeder S Krüger N Herrler T Erichsen S. SARS-CoV-2 cell entry depends on ACE2 and TMPRSS2 and is blocked by a clinically proven protease inhibitor. Cell. (2020) 181:271–80.e8. 10.1016/j.cell.2020.02.052 32142651 PMC7102627

[4] Richardson S Hirsch JS Narasimhan M Crawford JM McGinn T Davidson KW. Presenting characteristics, comorbidities, and outcomes among 5700 patients hospitalized with COVID-19 in the New York City area. JAMA. (2020) 323:2052–9. 10.1001/jama.2020.6775 32320003 PMC7177629

[5] Gilotra NA Minkove N Bennett MK Tedford RJ Steenbergen C Judge DP. Lack of relationship between serum cardiac troponin I level and giant cell myocarditis diagnosis and outcomes. J Cardiac Fail. (2016) 22:583–5. 10.1016/j.cardfail.2015.12.022 26768222

[6] Hu H Ma F Wei X Fang Y. Coronavirus fulminant myocarditis treated with glucocorticoid and human immunoglobulin. Eur Heart J. (2020) 4:260. 10.1093/eurheartj/ehaa190 32176300 PMC7184348

[7] Inciardi RM Lupi L Zaccone G Italia L Raffo M Tomasoni D. Cardiac involvement in a patient with coronavirus disease 2019 (COVID-19). JAMA Cardiol. (2020) 5:819–24. 10.1001/jamacardio.2020.1096 32219357 PMC7364333

[8] Zeng F Huang Y Guo Y Yin M Chen X Xiao L. Association of inflammatory markers with the severity of COVID-19: A meta-analysis. Int J Infect Dis. (2020) 96:467–74. 10.1016/j.ijid.2020.05.055 32425643 PMC7233226

[9] Rali AS Ranka S Shah Z Sauer AJ. Mechanisms of myocardial injury in coronavirus disease 2019. Card Fail Rev. (2020) 6:e15. 10.15420/cfr.2020.10 32537248 PMC7277776

[10] Chen C Zhou Y Wang DW. SARS-CoV-2: a potential novel etiology of fulminant myocarditis. Herz. (2020) 45:230–2. 10.1007/s00059-020-04909-z 32140732 PMC7080076

[11] Cooper LT Baughman KL Feldman AM Frustaci A Jessup M Kuhl U. The role of endomyocardial biopsy in the management of cardiovascular disease. J Am Coll Cardiol. (2007) 50:1914–31. 10.1016/j.jacc.2007.09.008 17980265

[12] Kostakou PM Kostopoulos VS Tryfou ES Giannaris VD Rodis IE Olympios CD. Subclinical left ventricular dysfunction and correlation with regional strain analysis in myocarditis with normal ejection fraction. A new diagnostic criterion. Int J Cardiol. (2018) 259:116–21. 10.1016/j.ijcard.2018.01.058 29579586

[13] Han J Mou Y Yan D Zhang Y-T Jiang T-A Zhang Y-Y. Transient cardiac injury during H7N9 infection. Eur J Clin Invest. (2015) 45:117–25. 10.1111/eci.12386 25431304

[14] Awadalla M Mahmood SS Groarke JD Hassan MZO Nohria A Rokicki A. Global longitudinal strain and cardiac events in patients with immune checkpoint inhibitor-related myocarditis. J Am College Cardiol. (2020) 75:467–78. 10.1016/j.jacc.2019.11.049 32029128 PMC7067226

[15] Sörensen J Harms HJ Aalen JM Baron T Smiseth OA Flachskampf FA. Myocardial efficiency. JACC: Cardiovasc Imag. (2019) 13:1564–76. 10.1016/j.jcmg.2019.08.030 31864979

[16] Chan J Edwards NFA Khandheria BK Shiino K Sabapathy S Anderson B. A new approach to assess myocardial work by non-invasive left ventricular pressure–strain relations in hypertension and dilated cardiomyopathy. Eur Heart J Cardiovasc Imaging. (2019) 20:31–9. 10.1093/ehjci/jey131 30247622

[17] Edwards NFA Scalia GM Shiino K Sabapathy S Anderson B Chamberlain R. Global myocardial work is superior to global longitudinal strain to predict significant coronary artery disease in patients with normal left ventricular function and wall motion. J Am Soc Echocardiogr. (2019) 32:947–57. 10.1016/j.echo.2019.02.014 31043359

[18] Lang RM Badano LP Mor-Avi V Afilalo J Armstrong A Ernande L. Recommendations for cardiac chamber quantification by echocardiography in adults: an update from the American society of echocardiography and the European association of cardiovascular imaging. J Am Soc Echocardiogr. (2015) 28:1–39.e14. 10.1016/j.echo.2014.10.003 25559473

[19] Nagueh SF Smiseth OA Appleton CP Byrd BF Dokainish H Edvardsen T. Recommendations for the evaluation of left ventricular diastolic function by echocardiography: an update from the American society of echocardiography and the European association of cardiovascular imaging. J Am Soc Echocardiograp. (2016) 29:277–314. 10.1016/j.echo.2016.01.011 27037982

[20] Kirkpatrick JN Mitchell C Taub C Kort S Hung J Swaminathan M. ASE statement on protection of patients and echocardiography service providers during the 2019 novel coronavirus outbreak. J Am College Cardiol. (2020) 75:3078–84. 10.1016/j.jacc.2020.04.002 32272153 PMC7194625

[21] Farsalinos KE Daraban AM Ünlü S Thomas JD Badano LP Voigt J-U. Head-to-head comparison of global longitudinal strain measurements among nine different vendors. J Am Soc Echocardiograp. (2015) 28:1171–81.e2. 10.1016/j.echo.2015.06.011 26209911

[22] Haji K Marwick TH. Clinical utility of echocardiographic strain and strain rate measurements. Curr Cardiol Rep. (2021) 23:18. 10.1007/s11886-021-01444-z 33594493

[23] D'Elia N Caselli S Kosmala W Lancellotti P Morris D Muraru D. Normal global longitudinal strain. JACC: Cardiovasc Imaging. (2020) 13:167–9. 10.1016/j.jcmg.2019.07.020 31481298

[24] Pieske B Tschöpe C de Boer RA Fraser AG Anker SD Donal E. How to diagnose heart failure with preserved ejection fraction: the HFA–PEFF diagnostic algorithm: a consensus recommendation from the heart failure association (HFA) of the European Society of Cardiology (ESC). Eur Heart J. (2019) 40:3297–317. 10.1093/eurheartj/ehz641 31504452

[25] Potter E Marwick TH. Assessment of left ventricular function by echocardiography. JACC: Cardiovascu Imaging. (2018) 11:260–74. 10.1016/j.jcmg.2017.11.017 29413646

[26] Russell K Eriksen M Aaberge L Wilhelmsen N Skulstad H Gjesdal O. Assessment of wasted myocardial work: a novel method to quantify energy loss due to uncoordinated left ventricular contractions. Am J Physiol Heart Circul Physiol. (2013) 305:H996–1003. 10.1152/ajpheart.00191.2013 23893165

[27] Russell K Eriksen M Aaberge L Wilhelmsen N Skulstad H Remme EW. A novel clinical method for quantification of regional left ventricular pressure-strain loop area: a non-invasive index of myocardial work. Eur Heart J. (2012) 33:724–33. 10.1093/eurheartj/ehs016 22315346 PMC3303715

[28] Hubert A Le Rolle V Leclercq C Galli E Samset E Casset C. Estimation of myocardial work from pressure-strain loops analysis: an experimental evaluation. Eur Heart J Cardiovasc Imaging. (2018) 19:1372–9. 10.1093/ehjci/jey024 29529181

[29] Manganaro R Marchetta S Dulgheru R Ilardi F Sugimoto T Robinet S. Echocardiographic reference ranges for normal non-invasive myocardial work indices: results from the EACVI NORRE study. Eur Heart J Cardiovasc Imaging. (2019) 20:582–90. 10.1093/ehjci/jey188 30590562

[30] El Mahdiui M van der Bijl P Abou R Ajmone Marsan N Delgado V Bax JJ. Global left ventricular myocardial work efficiency in healthy individuals and patients with cardiovascular disease. J Am Soc Echocardiograp. (2019) 32:1120–7. 10.1016/j.echo.2019.05.002 31279618

[31] Bouali Y Donal E Gallard A Laurin C Hubert A Bidaut A. Prognostic usefulness of myocardial work in patients with heart failure and reduced ejection fraction treated by sacubitril/valsartan. Am J Cardiol. (2020) 125:1856–62. 10.1016/j.amjcard.2020.03.031 32305222

[32] Abou R Leung M Khidir MJH Wolterbeek R Schalij MJ Ajmone Marsan N. Influence of aging on level and layer-specific left ventricular longitudinal strain in subjects without structural heart disease. Am J Cardiol. (2017) 120:2065–72. 10.1016/j.amjcard.2017.08.027 28951022

[33] Liu J-H Chen Y Yuen M Zhen Z Chan CW-S Lam KS-L. Incremental prognostic value of global longitudinal strain in patients with type 2 diabetes mellitus. Cardiovasc Diabetol. (2016) 15:22. 10.1186/s12933-016-0333-5 26842466 PMC4738770

[34] Wierzbowska-Drabik K Trzos E Kurpesa M Rechciński T Miśkowiec D Cieślik-Guerra U. Diabetes as an independent predictor of left ventricular longitudinal strain reduction at rest and during dobutamine stress test in patients with significant coronary artery disease. Eur Heart J Cardiovasc Imaging. (2018) 19:1276–86. 10.1093/ehjci/jex315 29236974

[35] Vrettos A Dawson D Grigoratos C Nihoyannopoulos P. Correlation between global longitudinal peak systolic strain and coronary artery disease severity as assessed by the angiographically derived SYNTAX score. Echo Res Pract. (2016) 3:29–34. 10.1530/ERP-16-0005 27248153 PMC4989094

[36] Liou K Negishi K Ho S Russell EA Cranney G Ooi S-Y. Detection of obstructive coronary artery disease using peak systolic global longitudinal strain derived by two-dimensional speckle-tracking: a systematic review and meta-analysis. J Am Soc Echocardiograp. (2016) 29:724–35.e4. 10.1016/j.echo.2016.03.002 27155815

[37] Smiseth OA Torp H Opdahl A Haugaa KH Urheim S. Myocardial strain imaging: how useful is it in clinical decision making? Eur Heart J. (2016) 37:1196–207. 10.1093/eurheartj/ehv529 26508168 PMC4830908

[38] Thavendiranathan P Poulin F Lim K-D Plana JC Woo A Marwick TH. Use of myocardial strain imaging by echocardiography for the early detection of cardiotoxicity in patients during and after cancer chemotherapy. J Am College Cardiol. (2014) 63:2751–68. 10.1016/j.jacc.2014.01.073 24703918

[39] Stokke TM Hasselberg NE Smedsrud MK Sarvari SI Haugaa KH Smiseth OA. Geometry as a confounder when assessing ventricular systolic function. J Am College Cardiol. (2017) 70:942–54. 10.1016/j.jacc.2017.06.046 28818204

[40] Honce R Schultz-Cherry S. Impact of obesity on influenza A virus pathogenesis, immune response, and evolution. Front Immunol. (2019) 10:1071. 10.3389/fimmu.2019.01071 31134099 PMC6523028

[41] Estabragh ZR Mamas MA. The cardiovascular manifestations of influenza: A systematic review. Int J Cardiol. (2013) 167:2397–403. 10.1016/j.ijcard.2013.01.274 23474244

[42] Cummings MJ Baldwin MR Abrams D Jacobson SD Meyer BJ Balough EM. Epidemiology, clinical course, and outcomes of critically ill adults with COVID-19 in New York City: a prospective cohort study. Lancet. (2020) 395:1763–70. 10.1016/S0140-6736(20)31189-2 32442528 PMC7237188

[43] Li Y Li H Zhu S Xie Y Wang B He L. Prognostic value of right ventricular longitudinal strain in patients with COVID-19. JACC: Cardiovasc Imaging. (2020) 13:2287–99. 10.1016/j.jcmg.2020.04.014 32654963 PMC7195441

[44] Argulian E Sud K Vogel B Bohra C Garg VP Talebi S. Right ventricular dilation in hospitalized patients with COVID-19 infection. JACC: Cardiovasc Imaging. (2020) 13:2459–61. 10.1016/j.jcmg.2020.05.010 32426088 PMC7228729

[45] Atri D Siddiqi HK Lang J Nauffal V Morrow DA Bohula EA. COVID-19 for the cardiologist: a current review of the virology, clinical epidemiology, cardiac and other clinical manifestations and potential therapeutic strategies. JACC: Basic Transl Sci. (2020) 5:518–36. 10.1016/j.jacbts.2020.04.002 32292848 PMC7151394

[46] Buja LM Wolf DA Zhao B Akkanti B McDonald M Lelenwa L. The emerging spectrum of cardiopulmonary pathology of the coronavirus disease 2019 (COVID-19): report of 3 autopsies from Houston, Texas, and review of autopsy findings from other United States cities. Cardiovasc Pathol. (2020) 48:107233. 10.1016/j.carpath.2020.107233 32434133 PMC7204762

[47] The GBD 2015 Obesity Collaborators. Health effects of overweight and obesity in 195 countries over 25 years. N Engl J Med. (2017) 377:13–27. 10.1056/NEJMoa1614362 28604169 PMC5477817

[48] Kass DA Duggal P Cingolani O. Obesity could shift severe COVID-19 disease to younger ages. Lancet. (2020) 395:1544–5. 10.1016/S0140-6736(20)31024-2 32380044 PMC7196905

[49] Pathan N Hemingway CA Alizadeh AA Stephens AC Boldrick JC Oragui EE. Role of interleukin 6 in myocardial dysfunction of meningococcal septic shock. Lancet. (2004) 363:203–9. 10.1016/S0140-6736(03)15326-3 14738793

[50] Johnson DE O'Keefe RA Grandis JR. Targeting the IL-6/JAK/STAT3 signalling axis in cancer. Nat Rev Clin Oncol. (2018) 15:234–8. 10.1038/nrclinonc.2018.8 29405201 PMC5858971

[51] Liu B Li M Zhou Z Guan X Xiang Y. Can we use interleukin-6 (IL-6) blockade for coronavirus disease 2019 (COVID-19)-induced cytokine release syndrome (CRS)? J Autoimmun. (2020) 111:102452. 10.1016/j.jaut.2020.102452 32291137 PMC7151347

[52] Huang C Wang Y Li X Ren L Zhao J Hu Y. Clinical features of patients infected with 2019 novel coronavirus in Wuhan, China. Lancet. (2020) 395:497–506. 10.1016/S0140-6736(20)30183-5 31986264 PMC7159299

[53] Chen N Zhou M Dong X Qu J Gong F Han Y. Epidemiological and clinical characteristics of 99 cases of 2019 novel coronavirus pneumonia in Wuhan, China: a descriptive study. Lancet. (2020) 395:507–13. 10.1016/S0140-6736(20)30211-7 32007143 PMC7135076

[54] Gao Y Li T Han M Li X Wu D Xu Y. Diagnostic utility of clinical laboratory data determinations for patients with the severe COVID-19. J Med Virol. (2020) 92:791–6. 10.1002/jmv.25770 32181911 PMC7228247

