# Supplementary material for: Myocardial Work Efficiency, A Novel Measure of Myocardial Dysfunction, Is Reduced in COVID-19 Patients and Associated With In-Hospital Mortality
Source: Front Cardiovasc Med. 2021 Jun 14;8:667721. doi: 10.3389/fcvm.2021.667721 (PMC8236710; doi:10.3389/fcvm.2021.667721)
Supplement: Supplementary file 1 [file Table_1.docx]

Supplementary Table 1. Value ranges of each inflammatory marker per tertile

| Inflammatory Marker | Tertile 1 | Tertile 2 | Tertile 3 |
| --- | --- | --- | --- |
| Interleukin-6, pg/mL | 0-68.3 | 69-212.17 | 213-2239.3 |
| Troponin I, ng/mL | 0.00-0.03 | 0.04-0.04 | 0.05-19.72 |
| Ferritin, ng/mL | 17-492 | 494-1075 | 1112-63425 |
| C-reactive protein, mg/dL | 0.00-7.4 | 7.9-23.2 | 27.5-292.3 |
| D-Dimer, mg/L | 0.29-1.07 | 1.1-3.92 | 4-31 |
| Fibrinogen, mg/dL | 90-509 | 511-622 | 633-1501 |

Supplementary Table 2. Subgroup Analyses of the Association of Mortality with GLS and MWE

|  | Normal LVEF | | Abnormal LVEF | |
| --- | --- | --- | --- | --- |
|  | Unadjusted | Adjusted | Unadjusted | Adjusted |
| GLS | 1.15 (0.94-1.40)  p=0.165 | 1.16 (0.94-1.43)  p=0.156 | 0.87 (0.63-1.21)  p=0.419 | 0.87 (0.58-1.29)  p=0.479 |
| MWE | 0.89 (0.78-1.00)  p=0.050 | 0.85 (0.74-0.99) **p=0.038** | 0.95 (0.85-1.07)  p=0.412 | 0.91 (0.78-1.07)  p=0.246 |
| LV EF | 1.06 (0.97-1.16)  p=0.205 | 1.07 (0.98-1.18)  p=0.144 | 0.98 (0.89-1.07)  p=0.612 | 0.98 (0.88-1.08)  p=0.621 |
|  | ARDS Absent | | ARDS Present | |
|  | Unadjusted | Adjusted | Unadjusted | Adjusted |
| GLS | 0.98 (0.75-1.28)  p=0.892 | 0.98 (0.70-1.37)  p=0.893 | 1.07 (0.91-1.26)  p=0.382 | 1.07 (0.91-1.26)  p=0.414 |
| MWE | 0.88 (0.72-1.06)  p=0.185 | 0.88 (0.68-1.12)  p=0.288 | 0.94 (0.86-1.03)  p=0.177 | 0.91 (0.82-1.01)  p=0.081 |
| LV EF | 1.07 (0.95-1.21)  p=0.280 | 1.07 (0.94-1.20)  p=0.304 | 0.99 (0.95-1.03)  p=0.516 | 0.99 (0.95-1.03)  p=0.685 |

*Adjusted for age and sex only
